# *Clostridioides difficile* and neurological disorders: New perspectives

**DOI:** 10.3389/fnins.2022.946601

**Published:** 2022-09-20

**Authors:** Manuele Biazzo, Manuela Allegra, Gabriele Deidda

**Affiliations:** ^1^The BioArte Limited, Life Sciences Park, San Gwann, Malta; ^2^SienabioACTIVE, University of Siena, Siena, Italy; ^3^Neuroscience Institute, National Research Council (IN-CNR), Padua, Italy; ^4^Department of Biomedical Sciences, University of Padua, Padua, Italy

**Keywords:** *C. difficile*, brain diseases, microbiota, gut-brain, fecal microbiota transplantation

## Abstract

Despite brain physiological functions or pathological dysfunctions relying on the activity of neuronal/non-neuronal populations, over the last decades a plethora of evidence unraveled the essential contribution of the microbial populations living and residing within the gut, called gut microbiota. The gut microbiota plays a role in brain (dys)functions, and it will become a promising valuable therapeutic target for several brain pathologies. In the present mini-review, after a brief overview of the role of gut microbiota in normal brain physiology and pathology, we focus on the role of the bacterium *Clostridioides difficile*, a pathogen responsible for recurrent and refractory infections, in people with neurological diseases, summarizing recent correlative and causative evidence in the scientific literature and highlighting the potential of microbiota-based strategies targeting this pathogen to ameliorate not only gastrointestinal but also the neurological symptoms.

## Introduction

The brain is an organ composed of neuronal and non-neuronal cell populations extremely interconnected in complex structural and functional networks that generate and control our behaviors from perception to motor action. Being so complex, it does not come as a surprise that a number of human pathological conditions involve in fact brain dysfunctions. Although genetic and experience-dependent actors surely control, modulate, and influence brain physiology and pathology, over the last decades it became clear that the brain is not alone. Starting from the pioneering studies on microbial ecology back in the 70s (Savage, 1977), the following scientific research on the *microbiome* (or biome) shed light and revealed the essential contribution of the microbial population living within the human body to the general physiological functions and health of an individual (Liang et al., 2018). In particular, those living and residing in the gastrointestinal tract (GIT) are referred to as *gut microbiota*, weights in total approximately akin to the human brain and communicating with the latter *via* the gut–brain axis where (i) microbial signals and metabolites are transmitted across the intestinal epithelium and *via* different pathways such as the trimethylamine (TMA)/trimethylamine N-oxide (TMAO), the short-chain fatty acids (SCFAs), and the primary and secondary bile acid (BAs) pathways; (ii) the *vagus nerve* acts as communication pathway (Rhee et al., 2009; Grenham et al., 2011); (iii) moreover, bacteria themselves are able to synthesize and then regulate the level of many neurotransmitters in the brain including major (GABA and glutamate) and neuromodulatory ones (serotonin, dopamine, and norepinephrine) (Desbonnet et al., 2010; Yano et al., 2015; Liu et al., 2017; Strandwitz et al., 2019). For these reasons, the gut microbiota and the brain influence each other *via* a bidirectional axis (the gut–brain axis), where the gut microbiota can exert control on brain functions in normal physiological conditions (from neurodevelopment till adulthood) and contribute to neurodevelopmental disorders (i.e., autism, schizophrenia, and Rett syndrome) and major brain diseases (*i.e*., Alzheimer's and Parkinson's diseases) (Grenham et al., 2011; Biagi et al., 2014; Mulak and Bonaz, 2015; Strati et al., 2016; Bonfili et al., 2017; Borghi et al., 2017; Vogt et al., 2017; Scheperjans et al., 2018; Winter et al., 2018; Ma et al., 2019; Deidda and Biazzo, 2021).

## Brain and gut brain: From neurophysiology and neuropathology to microbiota-based perspectives

Prenatally (*in utero*), the microbiota derived from the mother can influence (i) fetal brain development by means of metabolites reaching the fetus (Gomez De Aguero et al., 2016; Li et al., 2020), (ii) the development of the brain–blood barrier (Braniste et al., 2014), (iii) brain microglia cells (Thion et al., 2018), and (vi) thalamo-cortical axons growth (Vuong et al., 2020). Early in the postnatal life, the microbial colonization of the infant gut, who inherits the own microbiota *via* the first swallow and breastfeeding, keeps playing a role in normal brain development and maturation of adult brain functions. It has been demonstrated that animals with absent microbiota throughout life (so-called germ-free animals) show alterations in cognition, social behavior, and stress response in the adult life (Cryan and Dinan, 2012). Later in life, the gut microbiota can influence complex brain functions, such as social interactions by acting on the nutritional behavior of individual animals (Pasquaretta et al., 2018) and regulating pain, anxiety, mood and cognition (Cryan and Dinan, 2012). In mice, administration of *Campylobacter jejuni* triggers anxiety-like behaviors through a vagal-mediated pathway (Goehler et al., 2005). Patients with inflammatory bowel diseases display altered (i) microbial diversity and (ii) anxiety and depression behaviors (Barberio et al., 2021; Dubinsky et al., 2021).

Defective brain developments had been associated with a number of neurodevelopmental disorders, including, among others, autism spectrum disorder (ASD), schizophrenia spectrum disorders and Down syndrome (Di Cristo, 2007; Deidda et al., 2014, 2021). ASD is characterized by deficits in social communication, social interaction and restricted/repetitive behavioral patterns. GIT symptoms represent common comorbidity in ASD (Molloy and Manning-Courtney, 2003) and can worsen irritability and self-injury in patients (Carr and Owen-Deschryver, 2007). GIT symptom severity increases with the severity of ASD symptoms (Wang et al., 2011; Kho and Lal, 2018). Navarro et al. (2016) described that children with ASD represent a high percentage of patients that reports also GIT dysfunctions (Navarro et al., 2016). Apart from GIT symptoms, ASD is characterized also by an altered gut microbiota profile (Cao et al., 2013) showing higher levels of *Desulfovibrio* species, *Bacteroides vulgatus, and Clostridium bolteae* (Finegold et al., 2010; Wang et al., 2012; Pequegnat et al., 2013). Schizophrenia spectrum disorders are characterized by positive symptoms, negative symptoms, and cognitive symptoms. Gut microbiota alteration was described in several clinical trials finding an increase in *Lactobacillus, Saccharophagus, Ochrobactrum, Tropheryma, Halothiobacillus, Deferribacte*r, and *Halorubrum* (at the genus level) (Schwarz et al., 2018). These trials found also a decrease in *Anabaena, Nitrosospira*, and *Gallionella* (at the genus level) (Schwarz et al., 2018) and an increase in *Candida albicans* fungal species specifically in males (Severance et al., 2016). In Rett syndrome, an X chromosome-linked dominant neurodevelopmental disorder sharing features with ASD, GIT and nutritional problems are common (Motil et al., 2012), as well gut microbiota dysbiosis in terms of relative abundances of both bacterial and fungal components (Strati et al., 2016). Also, people with Down syndrome, the most frequent genetic cause of intellectual disability caused by the trisomy of human chromosome 21 (Bittles et al., 2007; Dierssen, 2012; Deidda et al., 2015; Contestabile et al., 2017), show gut microbiota dysbiosis with differences in *Parasporobacterium* spp. and *Sutterella* spp, *Veillonellaceae* (Biagi et al., 2014).

Not only in neurodevelopmental disorders but gut microbiota dysbiosis was widely reported also in major neurodegenerative diseases. For example, Alzheimer's disease is the first most common form of dementia in elderly life characterized by irreversible neurodegeneration impacting learning and memory functions (Wimo et al., 2017). In this pathology, the gut microbiota composition shows a decreased number of *Firmicutes* and *Actinobacteria* and an increased number of bacteria belonging to the Proteobacteria and Bacteroidetes phyla (Vogt et al., 2017). Strikingly, microbiota alterations might arise earlier than Alzheimer's clinical symptoms (Li et al., 2019).

Parkinson's disease is the second most common neurodegenerative disorder of aging (Disease et al., 2016), it results primarily from the death of dopaminergic neurons in the *substantia nigra pars compacta* in the basal ganglia, and it impacts motor movements (Moore et al., 2005; Antony et al., 2013). GIT symptoms are common including abdominal pain, bloating, and constipation (Abbott et al., 2001; Fasano et al., 2015; Georgescu et al., 2016; Santos et al., 2019). Also, gut microbiome dysfunctions are common (Hamano et al., 1993; Clairembault et al., 2015; Hasegawa et al., 2015; Keshavarzian et al., 2015; Klingelhoefer and Reichmann, 2015; Mulak and Bonaz, 2015; Unger et al., 2016; Bedarf et al., 2017; Hill-Burns et al., 2017; Parashar and Udayabanu, 2017; Scheperjans et al., 2018; Sun and Shen, 2018; Breen et al., 2019; Mihaila et al., 2019; Santos et al., 2019) with changes in the gut bacterial abundances of microbes (such as *Prevotellaceae* and *Enterobacteriaceae*) (Scheperjans et al., 2015), reduced content of *Dorea, Bacteroides, Prevotella, Faecalibacterium, Bacteroides massiliensis, Stoquefichus massiliensis, Bacteroides coprocola, Blautia glucerasea, Dorea longicatena, Bacteroides dorei, Bacteroides plebeius, Prevotella copri, Coprococcus eutactus*, and *Ruminococcus callidus*, and an increased content of *Ruminococcus bromii, Christensenella, Catabacter, Lactobacillus, Oscillospira, Bifidobacterium, Christensenella minuta, Catabacter hongkongensis, Lactobacillus mucosae*, and *Papillibacter cinnamivorans* (Bedarf et al., 2017; Petrov et al., 2017). Thereby, a plethora of evidence indicates that gut microbiota dysfunctions populate brain pathologies in humans. These pieces of evidence are more correlative than causative; in fact, direct strong causative evidence is still lacking in the clinic scientific literature and requires more investigations.

As far as the mechanisms underlying the link between the gut microbiota and neurodegenerative disorders (Parkinson's and Alzheimer's diseases) are concerned, these include gut microbiota-induced chronic inflammation, autoimmune dysregulation, protein misfolding, and defective protein clearance (Padhi et al., 2022). The gut dysbiosis would ultimately be responsible for triggering immune cell activation leading to the disruption of the intestinal and the blood–brain barrier permeability. In particular, the increased gut permeability would allow to pathogenic bacteria to infiltrate and release metabolites and endotoxins (e.g., the lipopolysaccharide, LPS) that can trigger chronic systemic inflammation and neurodegeneration (Brown, 2019).

The concept that gut microbiota affects also the brain and that brain–gut communication dysfunctions might play a role in the pathogenesis of neurological psychiatric disorders brought to a general interest into the search for new therapeutic solutions to tackle these pathologies, including prebiotics, synbiotics and probiotics (Wang et al., 2016; Markowiak and Slizewska, 2017; Deidda and Biazzo, 2021). For example, clinical studies exploited the use of probiotics in ameliorating the core symptoms of children with ASD: i) Kaluzna-Czaplinska and Blaszczyk (2012) showed that probiotic *Lactobacillus acidophilus* controls the level of D-arabinitol (a metabolite of most pathogenic *Candida* species) (Kaluzna-Czaplinska and Blaszczyk, 2012), ii) Shaaban et al. (2018) showed that probiotics *Lactobacillus acidophilus, Lactobacillus rhamnosus*, and *Bifidobacteria longum* improved the severity of autism and GIT symptoms (Shaaban et al., 2018), while iii) Santocchi et al. (2020) showed that the De Simone probiotic formulation (containing an eight-strain cocktail of probiotics belonging to *Lactobacillus, Bifidobacterium*, and *Streptococcus* genus) decreased the severity of ASD score in the group of children without GIT symptoms (Santocchi et al., 2020).

In clinical trials in people with Parkinson's disease, (i) *Lactobacillus casei shirota* improved stool consistency and defecation (Cassani et al., 2011), (ii) *Lactobacillus acidophilus* and *Bifidobacterium infantis* improved abdominal pain and bloating (Georgescu et al., 2016), and (iii) a probiotic mixture containing *Lactobacillus acidophilus, Lactobacillus reuteri, Bifidobacterium bifidum*, and *Lactobacillus fermentum* improved movement and metabolic parameters (Tamtaji et al., 2019). In animal models of Parkinson's disease, *Lacticaseibacillus rhamnosus* HA-114 improved hippocampal-dependent cognition deficits (Xie and Prasad, 2020).

In clinical trials in people with Alzheimer's disease, a mixed-species probiotic (including *Lactobacillus acidophilus, Lactobacillus casei, Bifidobacterium bifidum*, and *Lactobacillus fermentum*) improved the mini-mental state examination (MMSE) scores (Akbari et al., 2016). Recent meta-analysis studies showed an improvement in cognitive performance in people with Alzheimer's disease or mild cognitive impairment administered with probiotics (Den et al., 2020). In animal models of Alzheimer's disease, *Bifidobacterium breve* strain A1 prevented the cognitive dysfunctions induced by Aβ (Kobayashi et al., 2017), while Bonfili et al. (2017) showed that the SLAB51 probiotic formulation decreased the cognitive decline by means of a reduction in brain damage (Bonfili et al., 2017).

## *C. difficile* in brain pathologies

*Clostridioides difficile* (*C. difficile*) is an anaerobic gram-positive spore-forming bacterium emerging as an important pathogen in humans and being responsible for major infections in hospitalized patients (Eyre et al., 2013). It produces toxins (enterotoxin and cytotoxin which disrupts cytoskeleton signal transductions) that give rise to diarrhea, inflammation, dehydration, abdominal pain, loss of appetite, and nausea in infected patients (Di Bella et al., 2016). *C. difficile* eradication is difficult to achieve because of its high resistance to antibiotics that only recently begins to be elucidated (O'grady et al., 2021; Wickramage et al., 2021). Fecal microbiota transplantation (FMT), namely the transplant of fecal stools from healthy donors into infected patients (Zhang et al., 2018; Biazzo and Deidda, 2022), represents nowadays the tool used for the eradication of recurrent and refractory (where antibiotic treatment has failed) *C. difficile* infections in the GIT (Rossen et al., 2015), and also in other pathological conditions such as in ulcerative colitis and obesity (Carlucci et al., 2016).

As far as *C. difficile* in brain pathologies is concerned (Table 1), different studies found out a major dysbiosis in the gut microbiota composition in children with ASD in comparison with control (Finegold et al., 2002; Navarro et al., 2016) with an overrepresentation of *Clostridioides* species and underrepresentation of Bifidobacteria (Finegold et al., 2002; Song et al., 2004; Critchfield et al., 2011). The main limitation of these studies relies on the fact that pyrosequencing studies were exploited reporting alterations at the level of the *Clostridium* genus without verifying whether there is an actual alteration in the relative abundance of *C. difficile*.

**Table 1:** Summary of the studies related to the potential involvement of *C. difficile* in brain pathologies.

**Brain pathology**	**Main findings**	**Selected references**
Autism Spectrum Disorders (ASD)	Dysbiosis in the gut microbiota composition with an overrepresentation of *Clostridioides* species in autistic children in comparison to healthy control	Finegold et al., 2002; Song et al., 2004; Critchfield et al., 2011; Navarro et al., 2016
	Improvement of diarrhea, communication and behavioral symptoms upon vancomycin administration in children with severe ASD	Molloy and Manning-Courtney, 2003
Parkinson's disease	People within 2 years from the first diagnosis with *C. difficile* infection were at increased risk to develop the disease	Kang et al., 2020
Alzheimer's disease	Fecal microbiota transplantation improved gastrointestinal, cognitive and mood symptoms together with eradication of *C. difficile* in two patients	Hazan, 2020; Park et al., 2021
Multiple sclerosis	Fecal microbiota transplantation resolved *C. difficile* infection and improved constipation and neurological symptoms in four patients	Borody et al., 2011; Makkawi et al., 2018

The link between *C. difficile* infection and ASD is far to be elucidated. In mankind, administration of vancomycin, the drug of choice for *C. difficile* infections, improved diarrhea, but also communication and behavioral symptoms in children with severe ASD; however, these improvements regressed after drug withdrawal (Molloy and Manning-Courtney, 2003) suggesting that the *C. difficile* infection might be in part responsible. Studies in animal models found out that (i) mice treated with the short-chain fatty acid called propionic acid, a metabolite produced by *Clostridioides* species, develop autistic-like behaviors (Shultz et al., 2015), thereby identifying a possible causal link between the pathogen and ASD. However, propionic acid is produced by several taxa, not just *C. difficile*, thereby limiting the result of the study.

Concerning major neurodegenerative diseases, a Swedish population-based cohort study investigated whether *C. difficile* could represent a causative agent for Parkinson's disease by exploring the association between infection history and future disease risk. The study found out that people within 2 years from the first diagnosis with *C. difficile* infection were at increased risk to develop the disease in comparison with the group without the infection. Instead, in longer follow-up (more than 2 years), the infection was not associated with Parkinson's disease occurrence (Kang et al., 2020).

The link between *C. difficile* and brain pathologies has not been elucidated yet, but possible mechanisms recently come out in the spotlight. For example, it has been recently shown that *C. difficile* alters the metabolism of the neurotransmitter dopamine in the mouse brain, thus interfering with those cognitive functions that involve the neuromodulatory action of dopamine, such as motivation and memory consolidation in rodents (Vinithakumari et al., 2022). Thus, *C. difficile* might exert a control on the brain with a mechanism similar to those one exploited already by other micro-organisms that alter the level of neurotransmitters, for example, *Lactococcus* bacteria (Strandwitz et al., 2019), *Bifidobacterium infantis* (Desbonnet et al., 2010), or *Toxoplasma gondii* (Luder et al., 1999). Moreover, being *C. difficile* a pathogen synthetizing toxins, the mechanisms that ultimately lead impacting the brain might likely imply those already described for other bacteria which toxins can lead to a proinflammatory state (Brown, 2019).

## Microbiota-targeted strategies targeting *C. difficile* in brain pathologies

As far as microbiota-based strategies targeting *C. difficile* are concerned, when used to resolve infections caused by *C. difficile* in people with neurological diseases, FMT proved to be effective also in ameliorating neurological symptoms (Evrensel and Ceylan, 2016; Wortelboer et al., 2019; Vendrik et al., 2020; Xu et al., 2021).

Few clinical trials exploited FMT intervention in ASD and reported improvements in GIT and behavioral symptoms (Kang et al., 2017, 2019), although the authors did not specifically enroll children with ASD affected also by *C. difficile* infection.

Following a study in a mouse model of Alzheimer's disease showing positive results on Aβ and tau pathology obtained after FMT from control mice (Sun et al., 2019), Park et al. (2021) very recently exploited the FMT strategy in a person with Alzheimer's disease diagnosed with *C. difficile* infection. Since the classic antibiotic therapy failed in resolving the infection, the patient received two FMTs using fecal samples obtained from a healthy donor. After the transplantation, the author reported a general improvement of GIT symptoms together with elimination of *C. difficile* in the stools. More interestingly, to what it concerns the neurological aspect, the authors reported a general improvement in cognitive and mood symptoms (i.e., non-verbal learning, short-term memory and attention) (Park et al., 2021). Afterward, another work recapitulated the latter findings in a person with Alzheimer's disease who received FMT from his wife (Hazan, 2020). Altogether, these studies show that FMT might be taken into account when considering strategies aimed at efficiently tackling *C. difficile* infection and also improving GIT and cognitive deficits in people with Alzheimer's disease.

As far as multiple sclerosis is concerned, two studies explored FMT effects in four patients affected also by *C. difficile* infection (Borody et al., 2011; Makkawi et al., 2018). As reported in Alzheimer's disease, fecal stools derived from healthy donors resolved not only the infection caused by *C. difficile* but also improved constipation and neurological symptoms. Surprisingly, some patients showed also progressive improvement of leg paranesthesia and were eventually able to walk for long distances without help.

Antibiotic use is surely a primary risk factor for infection caused by *C. difficile*, but whether other medications could have an impact is not fully explored yet. In a recent study, Lalani et al. (2020) investigated in a group of US veterans whether the use of antidepressant and GABAergic drugs could contribute as a risk factor for the development of infections caused by *C. difficile*. The study found that antidepressant drug use was not correlated with risk of infection; instead, GABAergic drug use (benzodiazepines in particular) was associated (Lalani et al., 2020). *C. difficile* also been shown to result in severe brain infarction and thromboembolism (Kumar et al., 2021), but very few information is currently available and further investigations are needed to deeper study the correlation.

## Conclusion

Altogether, gut microbiota dysbiosis results in unbalanced microflora composition and plays a role in influencing brain functions and a number of brain pathologies (Deidda and Biazzo, 2021). In particular, *C. difficile* infection populates brain pathologies and the remarkable amelioration of clinical brain-related symptoms following *C. difficile* eradication in the gut could open up as a future perspective, the possibility of therapeutic interventions based on the exploitation of gut microbiota-targeted therapy for brain diseases to be used in the first place rather than being used as parallel therapy when also GIT dysfunctions are involved.

## Author contributions

GD and MB conceptualized and wrote the manuscript. MA contributed to writing the manuscript. All authors read and revised the manuscript.

## Conflict of interest

Author MB is CEO of the BioArte Ltd. The remaining authors declare that the research was conducted in the absence of any commercial or financial relationships that could be construed as a potential conflict of interest.

## Publisher's note

All claims expressed in this article are solely those of the authors and do not necessarily represent those of their affiliated organizations, or those of the publisher, the editors and the reviewers. Any product that may be evaluated in this article, or claim that may be made by its manufacturer, is not guaranteed or endorsed by the publisher.
